# Self-Rated Health and Semen Quality in Men Undergoing Assisted Reproductive Technology

**DOI:** 10.1001/jamanetworkopen.2023.53877

**Published:** 2024-01-30

**Authors:** Xiao-Ying Liu, Yan-Ling Deng, Pan-Pan Chen, Chong Liu, Yu Miao, Min Zhang, Fei-Peng Cui, Jia-Yue Zeng, Yang Wu, Cheng-Ru Li, Chang-Jiang Liu, Qiang Zeng

**Affiliations:** 1 Department of Occupational and Environmental Health, School of Public Health, Tongji Medical College, Huazhong University of Science and Technology, Wuhan, Hubei, PR China; 2 Key Laboratory of Environment and Health, Ministry of Education & Ministry of Environmental Protection, and State Key Laboratory of Environmental Health (incubating), School of Public Health, Tongji Medical College, Huazhong University of Science and Technology, Wuhan, Hubei, PR China; 3 NHC Key Laboratory of Birth Defects and Reproductive Health, Chongqing Population and Family Planning Science and Technology Research Institute, Chongqing, PR China

## Abstract

**Importance:**

Numerous studies have documented the association of self-rated health (SRH) with chronic diseases. However, few studies have investigated its association with semen quality.

**Objective:**

To examine the association of SRH with semen quality among men undergoing assisted reproductive technology (ART) in China.

**Design, Setting, and Participants:**

This cross-sectional study recruited male partners in couples undergoing ART treatment at the Center for Reproductive Medicine, Tongji Hospital, Wuhan, China. A total of 1262 men underwent 2 semen examinations and completed a questionnaire on SRH between December 2018 and January 2020. Data analysis was performed from November 20, 2022, to March 24, 2023.

**Exposure:**

SRH, including overall physical and mental health, as well as reproductive-related physical and mental health specifically, were reported at baseline recruitment.

**Main Outcomes and Measures:**

Sperm concentration, sperm progressive motility, sperm motility, and sperm count as semen quality parameters.

**Results:**

The study included 1262 men with a mean (SD) age of 32.79 (5.25) years and body mass index of 24.37 (3.68). Men with poorer SRH had lower semen quality (eg, sperm concentration among poor vs very good overall physical health: percentage variation, −14.67%; 95% CI, −23.62% to −4.66%). Among 4 components of SRH, a greater reduction in semen quality was estimated for reproductive-related SRH compared with overall SRH, whereas the greatest reduction was observed for reproductive-related physical SRH. In comparison with men with very good reproductive-related physical SRH, men with poor reproductive-related physical SRH had differences of −24.78% (95% CI, −32.71% to −15.93%) and −25.61% (95% CI, −33.95% to −16.22%) in sperm count and concentration, respectively, and regression coefficients of −9.38 (95% CI, −12.01 to −6.76) and −9.24 (95% CI, −11.82 to −6.66) for sperm motility and sperm progressive motility, respectively.

**Conclusions and Relevance:**

In this cross-sectional study of Chinese men, poorer SRH was associated with lower semen quality, and reproductive-related physical SRH was the most pronounced indicator. Our findings suggest that SRH, especially reproductive-related physical SRH, was a good indicator of semen quality, which should inform public and clinical regulatory decisions.

## Key Points

**Question:**

Is self-rated health (SRH) associated with semen quality among men undergoing assisted reproductive technology?

**Findings:**

In this cross-sectional study of 1262 men in China, those with poorer SRH had lower semen quality. A greater reduction in semen quality was estimated for reproductive-related SRH compared with overall SRH, whereas the greatest reduction was observed for reproductive-related physical SRH.

**Meaning:**

Our findings suggest that SRH, especially reproductive-related physical SRH, is a good indicator of semen quality, which should inform public and clinical regulatory decisions.

## Teaser

This cross-sectional study examines the association of self-rated health with semen quality among men undergoing assisted reproductive technology (ART) in China.

## Introduction

Available survey data estimate that the global prevalence of infertility is 8% to 12% in reproductive-aged couples, with half of the cases overall contributed by the male factor.^1^ Numerous factors are responsible for the increasing prevalence of male infertility, of which lower semen quality is a major contributor.^2^ Currently, accumulating evidence has shown a declining trend in semen quality worldwide over the last few decades.^3,4^ In China, a systematic review also concluded that sperm concentration and count decreased by 36.95% and 28.13%, respectively, among healthy men from 1981 to 2019.^5^ Understanding risk factors for decreasing semen quality has been an increasing global concern that can inform public health interventions for improving fertility. Environmental factors such as smoking^6^ and exposures to endocrine-disrupting chemicals^7,8^ have been associated with reduced semen quality. Furthermore, psychological factors have been proposed to play a role.^9,10^

Self-rated health (SRH) is an individual’s subjective assessment of their health, including their perceptions of health history, current health burden, and awareness of future health risks, in aspects of both physiology and psychology.^11,12,13,14^ Interest in the role of SRH as an indicator of health status has risen substantially over the past decades.^15^ Prospective studies have shown that SRH is a noninvasive and reliable key predictor for important health events such as cardiovascular health,^16^ mortality,^17^ and health services.^18^ Moreover, SRH is prioritized as an alternative health measure of overall health status when there are no or multiple objective health indicators assessed by clinical examinations.^19,20^ Therefore, SRH has been proposed as a simple and valid indicator for individual health events and certain clinical parameters.

Studies have also explored SRH as an indicator of human reproductive health. Numerous research studies have demonstrated the associations of poor SRH during pregnancy with more complications and adverse birth outcomes, such as gestational diabetes, preterm birth, and infant low birth weight.^15,19,21,22,23^ However, only limited studies have investigated the association of SRH with male reproductive health. Several studies have presented the associations between SRH and altered reproductive hormones in middle-aged and older men.^24,25,26^ Nevertheless, only 1 study of which we are aware has investigated the association between SRH and semen quality in young men.^27^ This study only relied on single semen measurement, which may bias the estimated associations due to within-individual variability in semen quality parameters.^28^ Moreover, most previous studies focused on overall SRH but did not differentially consider psychological and physical components and also did not capture reproductive-related SRH specifically. Human reproductive health involves both psychological and physical changes,^29^ and it is possible that SRH including those components, specifically in terms of reproductive-related components, would be superior.^30^

Among men attending an infertility center in Wuhan, China, we relied on 4 items of SRH involving overall physical and mental health as well as reproductive-related physical and mental health specifically. This study aimed to explore the 4 components of SRH in association with semen quality. Since body mass index (BMI) and age are predictors of semen quality,^31,32^ we additionally investigated potential modification by BMI and age on the associations between SRH and semen quality.

## Methods

### Study Population

The study population was from a prospective cohort Tongji Reproductive and Environmental (TREE) study, as detailed previously.^33^ Briefly, between December 2018 and January 2020, couples aged 20 years or older seeking assisted reproductive technology (ART) were enrolled at the Reproductive Medicine Center, Tongji Hospital, Wuhan, China. At baseline recruitment, each participant was asked to complete a face-to-face questionnaire after signing an informed consent form including basic characteristics, lifestyle habits, medical history, and SRH information. Among the 2045 men recruited, 1330 men who provided 2 semen samples and had available evaluation data for SRH were considered eligible for this study. We further excluded men with azoospermia (n = 4) or chromosomal abnormalities (n = 64), leaving 1262 men included. No significant differences in characteristics were observed between the whole population and the included population, except for abstinence time at second measurement (eTable 1 in Supplement 1). This study received approval from the ethics committee at Tongji Medical College, adhering to the Strengthening the Reporting of Observational Studies in Epidemiology (STROBE) reporting guideline for cross-sectional studies.

### Assessment of SRH

We evaluated SRH with a 4-item questionnaire involving overall physical and mental health as well as reproductive-related physical and mental health specifically. SRH was evaluated by the respondent’s answers to the 4 questions in the questionnaire at recruitment: “How would you rate your overall physical health status (overall mental health/reproductive-related physical health/ reproductive-related mental health)?” The answer choices were as follows: very good, good, neither good nor poor, poor, and very poor. Neither good nor poor is considered a deviation from good healthy normal state, trending nearer to poor than good health.^30,34^ Meanwhile, given that few participants in this study reported very poor or poor SRH, we merged the very poor, poor, and neither good nor poor into poor SRH. Such classification of SRH has been used in previous studies.^30,35,36^

### Semen Collection and Analysis

Each participant provided 2 semen samples via masturbation into a sterile plastic specimen cup on different days. The first one was on the day of baseline recruitment, and the second one was on the day of ART treatment, with median (IQR) intervals of 90 (63-143) days between the 2 sample collections. Semen collection and analysis were accomplished in compliance with the guidelines set forth by the World Health Organization^37^ as detailed in previous research.^38^ Briefly, the abstinence time was reported by the participants before semen collection. Next, analyses of sperm concentration and motility were conducted using a microcellular slide and computer-assisted semen test (CASA, WLJX 9000), and semen volume was calculated by weighing. Total sperm count equals sperm concentration multiplied by sperm volume, and total sperm motility equals progressive sperm motility plus nonprogressive sperm motility.^37^ All semen sample evaluations in this hospital were conducted by 2 professional technicians, and the measurable results revealed no statistically significant differences. External quality controls were also built by the semen laboratory and supervised by the Hubei Provincial Quality Control Center.

### Statistical Analysis

We conducted descriptive statistics for the basic characteristics, SRH levels, and semen quality parameters of the study population. Intraclass correlation coefficients (ICCs), defined as the intra-individual variability divided by the total variability, were calculated to assess the reproducibility of semen quality parameters using multilevel random-effects models. An ICC greater than 0.75 is defined as excellent; 0.40 to 0.75, fair to good; and less than 0.40, poor.^39^

A linear mixed-effects model adjusting for within-participant correlations by carrying a participant-specific random intercept was applied to explore the association between SRH and semen quality. SRH as an independent variable was categorized as poor, good, and very good, where very good SRH served as the reference group. The *P* values for trends across decreasing SRH groups were tested when presuming equivalent distances between poor, good, and very good SRH.^27^ Sperm concentration and count were transformed using natural logarithm (ln) to improve normality and were then back-transformed to get the percentage changes for an explanation of the result by using the equation: 100% × (exp[β] − 1).

Selection of potential confounders was derived from previous literature^40,41,42,43,44,45,46,47^ and then finalized by directed acyclic graphs.^48^ We finally retained these covariates in the adjusted model: BMI (calculated as weight in kilograms divided by height in meters squared), age, alcohol use, smoking status, income, education, and abstinence time (eFigure in Supplement 1). Age and BMI were treated as continuous variables; alcohol use (ever vs never) and education (<high school vs ≥high school) were considered dichotomous variables; smoking status (current and former vs never smoker) were modeled as dummy variables; abstinence time (3-5 days and >5 day vs <3 days) and income (<¥3000/mo, ¥3000-10 000/mo, and >¥10 000/mo [to convert to US dollars, multiply by 0.14]) were regarded as ordinal variables.

To assess robust findings, we first reanalyzed the data by excluding men with reproductive diseases (eg, varicocele, mumps, orchitis, epididymitis) (n = 143). Second, we excluded men using medication for spermatogenesis within the past 3 months (n = 145). Stratified analyses were also applied to examine whether age (<30 vs ≥30 years) and BMI (<24 vs ≥24) altered the associations between SRH and semen quality, and a cross-product term involving age or BMI and SRH were incorporated into the models to compute *P* values for interaction. Data analysis for this study was performed from November 20, 2022, to March 24, 2023. Statistical analyses were conducted using the SPSS software version 25.0 (IBM Corp) and R software version 4.0.2 (R Project for Statistical Computing). Statistical significance was set at a 2-sided *P* < .05.

## Results

Characteristics of study population are reported in Table 1. The mean (SD) age and BMI of all men were 32.79 (5.25) years and 24.37 (3.68), respectively. Among them, there were 909 men (72.0%) aged 30 years or older and 680 men (53.9%) with a BMI of 24 or greater. More than half of the men reported education of high school or greater (849 [67.3%]), an abstinence time of 3 to 5 days (863 [68.4%] at first measurement and 947 [75.0%] at second measurement), and no alcohol use (1006 [79.7%]). Among this study population, more than 40% of men reported poor overall and reproductive-related physical SRH, while less than 40% of men reported good overall and reproductive-related mental SRH. In terms of specific reproductive-related physical health, 606 (48.0%), 456 (36.1%), and 200 (15.8%) reported poor, good, and very good SRH, respectively.

**Table 1.** Characteristics of 1262 Male Participants in the Study

Characteristics	No. (%)
Age at recruitment, y	
Mean (SD)	32.79 (5.25)
<30	353 (28.0)
≥30	909 (72.0)
Ethnicity	
Han	1215 (96.3)
Other^a^	47 (3.7)
BMI	
Mean (SD)	24.37 (3.68)
<18.5	36 (2.9)
18.5-24.0	546 (43.3)
>24.0	680 (53.9)
Smoking status	
Never smoker	542 (42.9)
Former smoker	190 (15.1)
Current smoker	530 (42.0)
Alcohol use	
No	1006 (79.7)
Yes	256 (20.3)
Educational level	
<High school	413 (32.7)
≥High school	849 (67.3)
Household income, ¥/mo^b^	
<3000	133 (10.5)
3000-10 000	888 (70.4)
>10 000	241 (19.1)
Abstinence time at first measurement, d^c^	
<3	157 (12.4)
3-5	863 (68.4)
>5	232 (18.4)
Abstinence time at second measurement, d^d^	
<3	96 (7.6)
3-5	947 (75.0)
>5	176 (13.9)
Reproductive-related physical health	
Poor	606 (48.0)
Good	456 (36.1)
Very good	200 (15.8)
Reproductive-related mental health	
Poor	501 (39.7)
Good	512 (40.6)
Very good	249 (19.7)
Overall physical health	
Poor	538 (42.6)
Good	510 (40.4)
Very good	214 (17.0)
Overall mental health	
Poor	414 (32.8)
Good	568 (45.0)
Very good	280 (22.2)

Abbreviation: BMI, body mass index (calculated as weight in kilograms divided by height in meters squared).

^a^ Others included Bai, Dong, Hui, Man, Mongolian, Miao, Tujia, Uighur, Yao, Yi, and Zhuang ethnic groups.

^b^ To convert yuan to US dollars, multiply by 0.14.

^c^ A total of 10 participants were missing abstinence time at first measurement.

^d^ A total of 43 participants were missing abstinence time at second measurement.

Table 2 shows the distribution and reproducibility of semen quality parameters from 1262 study participants contributing 2524 semen samples. The median (range) values for sperm concentration, count, motility, and progressive motility were 45.0 (2.4-250.9) million/mL, 145.0 (1.4-1178.7) million/ejaculate, 48.0% (1.4%-90.8%) motile, and 45.0% (0.6%-86.7%) motile, respectively. The ICCs of sperm concentration, count, motility, and progressive motility in repeated semen samples were 0.5, 0.4, 0.5, and 0.5, respectively, indicating fair to good reliability. Men with poor SRH had apparently lower sperm concentration, sperm count, sperm motility, and sperm progressive motility compared with those with very good SRH (eTable 2 in Supplement 1).

**Table 2.** Distribution and Reproducibility of Semen Quality Parameters Among 1262 Male Participants

Variable	First measurement^a^			Second measurement^b^			Mean	Median	ICC
	Mean	Median	Range	Mean	Median	Range			
Sperm concentration, million/mL	55.0	47.4	2.4-250.9	50.7	43.0	3.0-233.0	52.9	45.0	0.5
Sperm count, million/ejaculate	195.1	168.7	1.4-1178.7	150.7	123.0	5.0-696.0	172.9	145.0	0.4
Sperm motility, % motile	51.2	52.0	1.4-90.8	43.0	44.5	2.0-86.0	47.1	48.0	0.5
Sperm progressive motility, % motile	48.3	48.7	0.6-86.7	39.9	41.0	1.0-84.0	44.1	45.0	0.5

Abbreviation: ICC, intraclass correlation coefficient.

^a^ A total of 17 samples were missing sperm progressive motility in the first measurement.

^b^ A total of 2 samples were missing sperm progressive motility and sperm count and 1 was missing sperm concentration in the second measurement.

**Figure 1.**
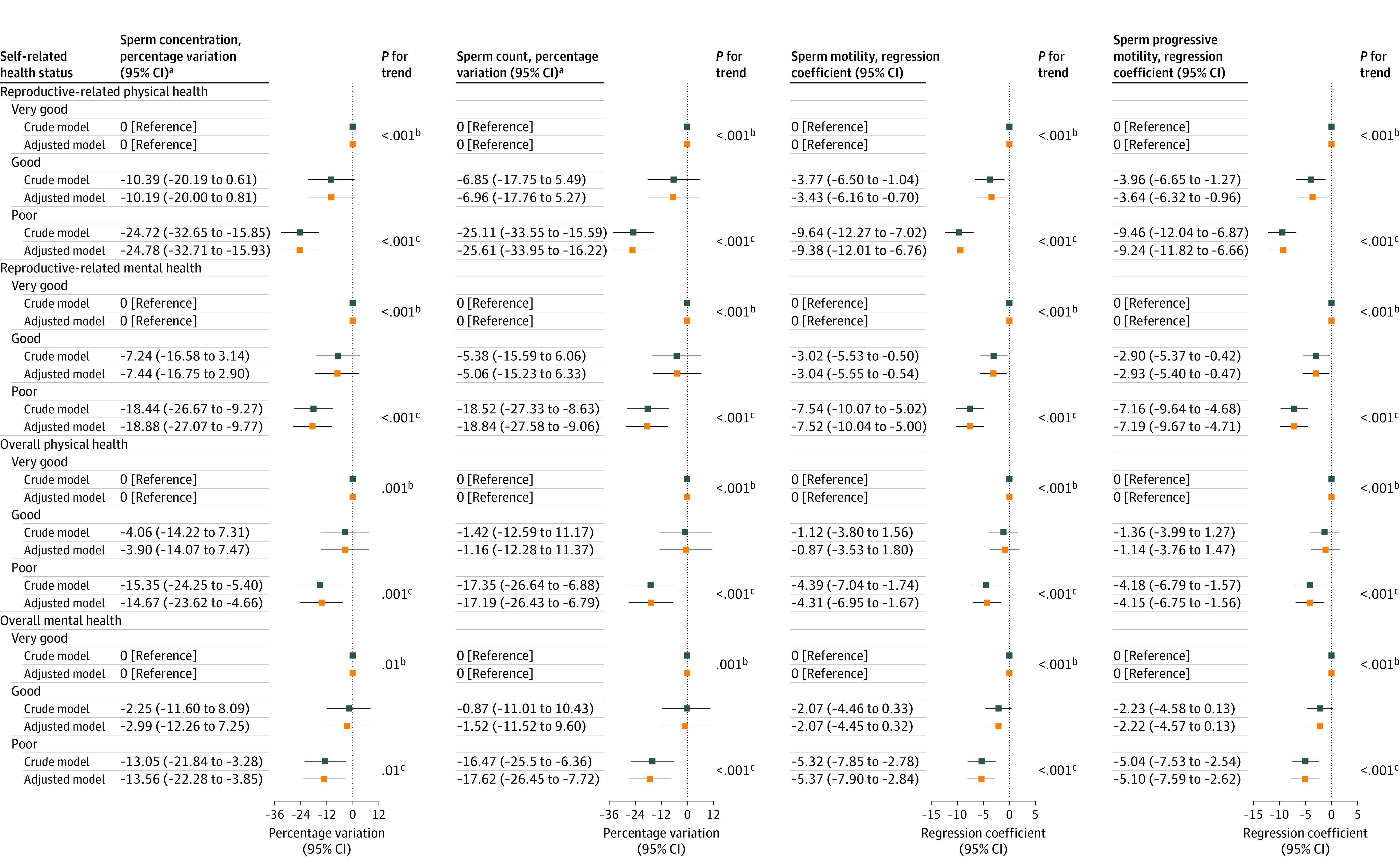
Percentage Variations and Regression Coefficients for Semen Quality Parameters Associated With Self-Rated Health Adjusted models were adjusted for age, body mass index, education, household income, alcohol use, abstinence time, and smoking status. ^a^Sperm concentration and count were natural-log transformed and back-transformed (100 × [exp{β} − 1]) to obtain percentage variation. ^b^*P* for trend in the crude model. ^c^*P *for trend in the adjusted model.

The association between SRH and semen quality from linear mixed-effects models is presented in Figure 1 and eTable 3 in Supplement 1. Poorer SRH was associated with lower semen quality in crude and adjusted models (eg, sperm concentration among poor vs very good overall physical health: percentage variation, −14.67%; 95% CI, −23.62% to −4,66%). The magnitude of the association between poorer reproductive-related SRH and lower semen quality was greater than that of overall SRH, and the greatest associations were estimated for reproductive-related physical SRH. In adjusted models, compared with the participants with very good reproductive-related physical SRH, men with poor reproductive-related physical SRH had differences of −24.78% (95% CI, −32.71% to −15.93%) and −25.61% (95% CI, −33.95% to −16.22%) in sperm concentration and count, respectively, and regression coefficients of −9.38 (95% CI, −12.01 to −6.76), and −9.24 (95% CI, −11.82 to −6.66) for sperm motility and progressive motility, respectively.

These associations between SRH and semen quality remained robust when we excluded men with reproductive diseases (eTable 4 in Supplement 1). After further excluding men using spermatogenesis medications within the past 3 months, we found that the magnitude of these associations was attenuated but mostly remained statistically significant, except for the associations of overall physical and mental SRH with sperm concentration and count as well as reproductive-related mental SRH with sperm count (eTable 5 in Supplement 1).

**Figure 2.**
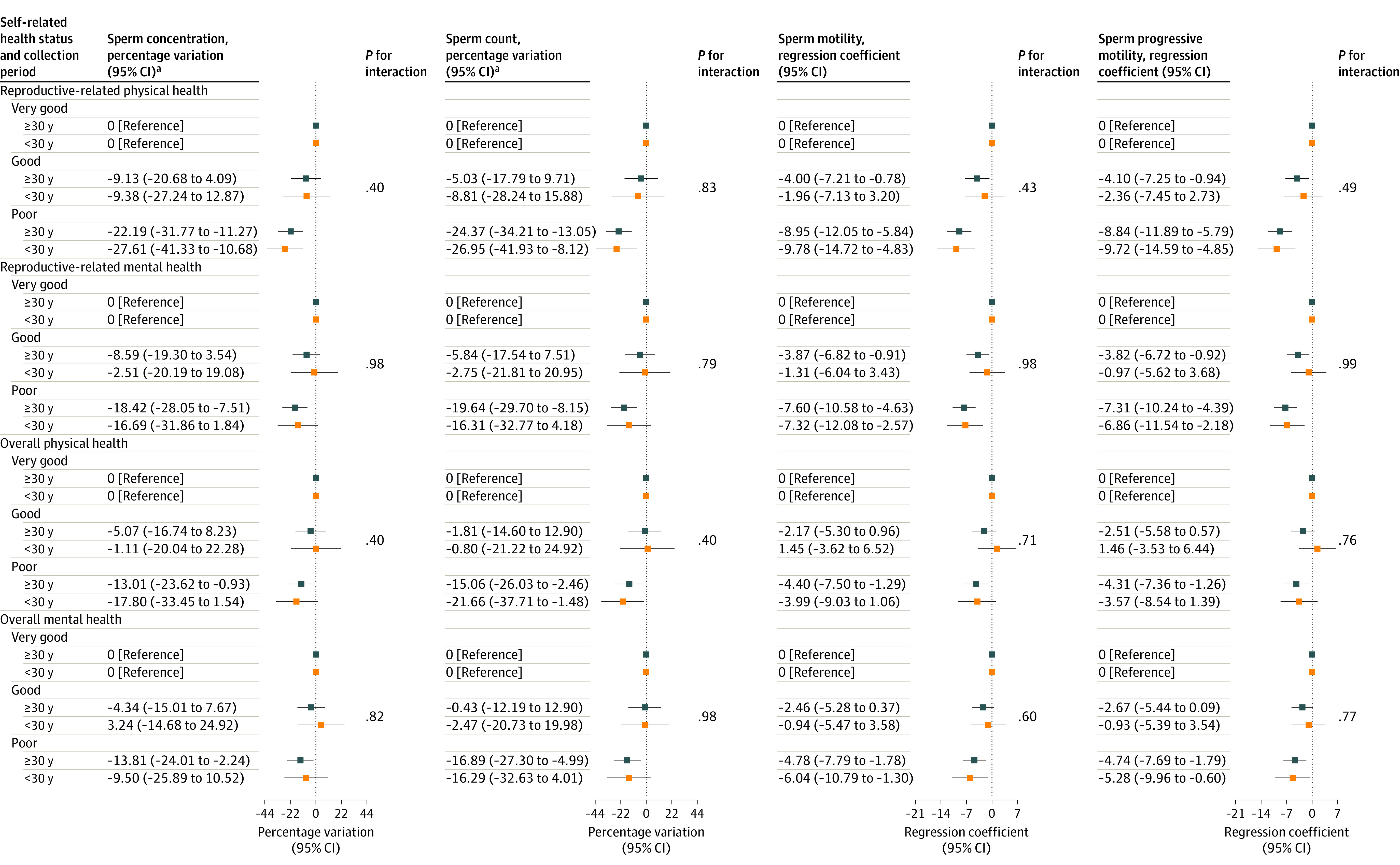
Percentage Variations and Regression Coefficients for Semen Quality Parameters Associated With Self-Rated Health Stratified by Age Models were adjusted for body mass index, education, household income, alcohol use, abstinence time, and smoking status. ^a^Sperm concentration and count were natural-log transformed and back-transformed (100 × [exp{β} − 1]) to obtain percentage variation.

**Figure 3.**
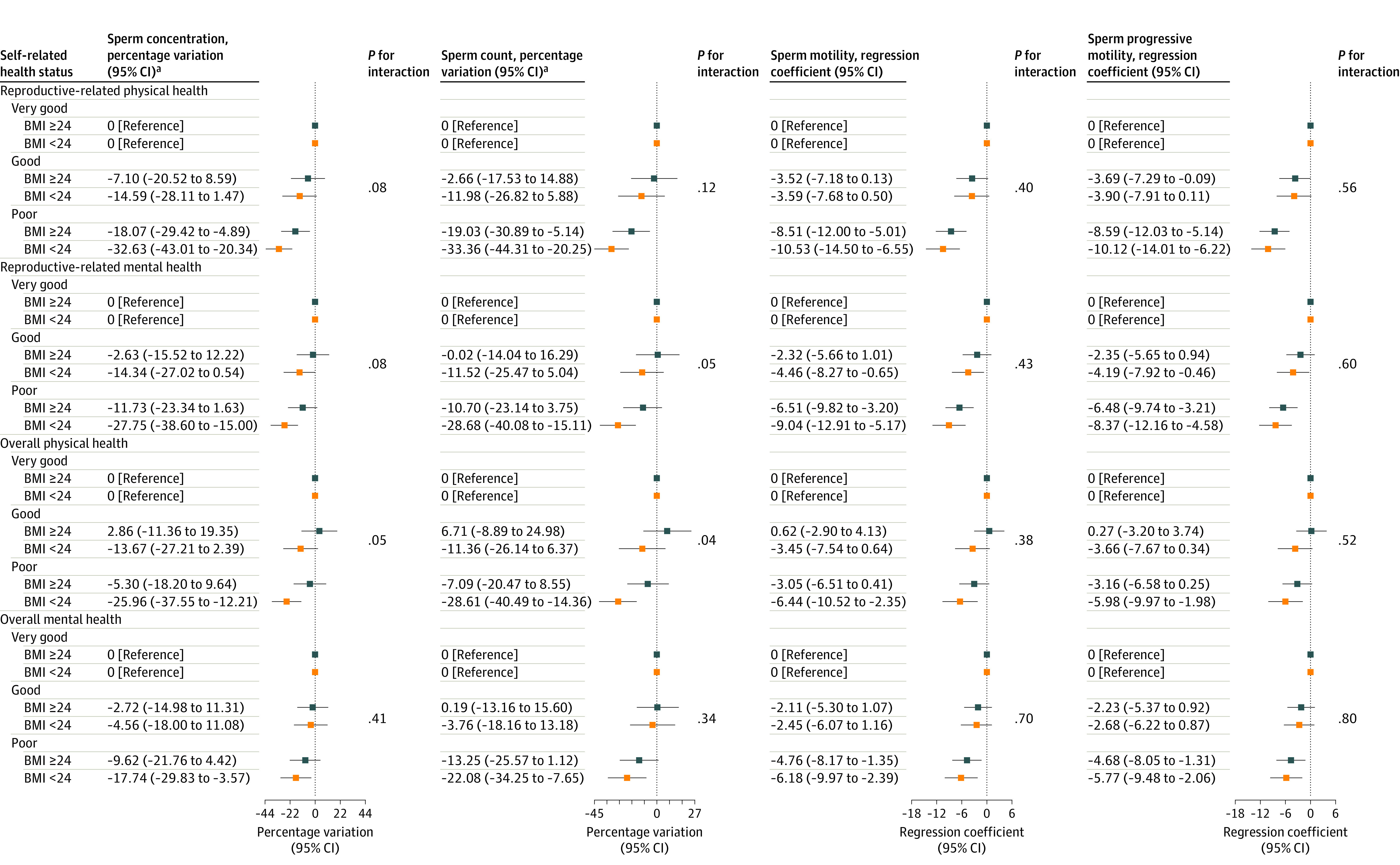
Percentage Variations and Regression Coefficients for Semen Quality Parameters Associated With Self-Rated Health Stratified by Body Mass Index (BMI) Models were adjusted for age, education, household income, alcohol use, abstinence time, and smoking status. ^a^Sperm concentration and count were natural-log transformed and back-transformed (100 × [exp{β} − 1]) to obtain percent variation.

Results from stratification analyses by age and BMI are shown in Figure 2 and Figure 3 as well as eTable 6 and eTable 7 in Supplement 1. These differences in the associations between SRH and semen quality stratified by age were not statistically significant. However, when stratified by BMI, the associations of poorer SRH with lower semen quality were stronger among men with BMIs less than 24. Specifically, these differences were statistically significant for reproductive-related mental SRH in relation to sperm count (eg, poor reproductive-related mental health: percentage variation, −28.68%; 95% CI, −40.08% to −15.11%) as well as for overall physical SRH in relation to sperm concentration and count (sperm concentration among those with poor overall physical health: percentage variation, −25.96%; 95% CI, −37.55% to −12.21%; sperm count among those with poor overall physical health: percentage variation: −28.61%; 95% CI, −40.49% to −14.36%).

## Discussion

Based on the data from the TREE cohort by using 4 items of SRH and repeated semen parameter measurements among men undergoing ART, we observed that poorer SRH was associated with lower semen quality. The magnitude of the association was greater in terms of reproductive-related SRH than that of overall SRH, and the greatest associations observed were for reproductive-related physical SRH. Moreover, we found that poorer SRH in association with lower semen quality was more pronounced among men with a BMI of less than 24.

Previous epidemiological studies have investigated the association between SRH and male reproductive health, mainly involving reproductive hormones, with conflicting findings. One study^25^ conducted among men aged 70 years or older in Australia examined the cross-sectional and longitudinal associations between SRH and reproductive hormones. The cross-sectional data showed that poorer SRH was associated with lower levels of serum testosterone, estrone, and calculated free testosterone, but longitudinal data only indicated a positive association between SRH and estrone.^25^ On the contrary, poor SRH was observed to be associated with higher serum estradiol levels in community-dwelling men aged 30 to 89 years from the Framingham Heart Study.^26^ A cross-sectional study of 466 elderly men in Finland^24^ reported null associations between SRH and sex hormones such as estradiol, testosterone, follicle-stimulating hormone, and luteinizing hormone after adjusting for age and BMI, but a positive association was observed with testosterone and free testosterone after adjusting for age. Male reproductive hormone levels play an essential role in maintaining normal spermatogenesis.^49^ Abnormal alterations in reproductive hormone levels may adversely affect on semen quality. In the current study, our findings observed that men with poorer SRH had decreased semen quality. Consistent with our results, a previous study also reported significant associations between poorer SRH and lower sperm concentration, morphologically normal sperm, and total sperm count among 3457 young Danish men attending a compulsory physical examination.^27^

In terms of specific SRH, our results suggested that reproductive-related SRH had stronger associations with semen quality compared with overall SRH. Such findings are expected because reproductive-related SRH focuses on the specific aspects of health that are most relevant to reproductive health, whereas overall SRH is a more general measure of health. Many studies have shown that reproductive-related clinical assessment indicators, such as altered reproductive hormone levels and erectile dysfunction, are strongly associated with SRH.^25,50,51^ Poorer SRH and subsequent worsening of SRH can be predicted by lower reproductive hormone levels over time among men,^25^ which means that men with declining reproductive system function will make a more pessimistic assessment of their health, resulting in low SRH ratings.

Although multiple terms of SRH, such as physical and mental SRH, are highly intercorrelated (eTable 8 in Supplement 1), they are not redundant measures of the same thing.^30,52^ Bodily sensations play a potentially important component pathway from the physiological state to SRH.^11^ The perception of bodily sensations, such as pain, impaired physical mobility, or sexual dysfunction, can significantly influence a personal subjective assessment of overall health status.^53,54^ Our participants might have noticed any potential abnormalities in their reproductive organs or any unusual reproductive-related symptoms because they were seeking ART treatment, although infertility could have been related to their female partners. Thus, those could explain our study’s findings on the largest association of reproductive-related physical SRH in association with semen quality among the 4 terms of SRH.

Interestingly, our findings showed that the associations of poorer SRH with lower semen quality were stronger among men with lower vs higher BMI. This suggests that semen quality among leaner men is more susceptible to SRH. Studies have found that individuals with normal weight are far from satisfied with their body shape or size, and thus, they have higher concerns about maintaining their weight or fear of gaining weight, which in turn can cause anxiety and lead to worse SRH.^55,56^ Moreover, it is possible that the effect of overweight or obesity on semen quality may weaken or mask the association between SRH and semen quality among men with higher BMIs. Obesity can impair semen quality by regulating fatty acid metabolism in the testes and increasing mitochondrial damage in spermatozoa to reduce sperm concentration, viability, and normal morphology.^57,58,59^

The biological mechanisms underlying the association between poorer SRH and lower semen quality have not been elucidated. It has been suggested that environmental stressors trigger an increase of glucocorticoids that can impair the hypothalamic-hypophysis-gonadic axis, resulting in reproductive dysfunction.^60,61,62^ The stress-induced glucocorticoids have been observed to produce apoptosis in mesenchymal cells and germ cells because of the presence of glucocorticoid receptors in both cells.^63^ Furthermore, animal studies have found that excess glucocorticoids decrease the testicular Leydig cell steroidogenic capacity, testosterone biosynthesis, and the number of Leydig cells per testis.^64,65,66,67^ Alterations in sex hormone levels may also be another mechanism involved in the adverse association of poorer SRH with semen quality.^68,69^ Primates exposed to acute and chronic social stresses have been found to alter the reproductive axis and give rise to suppression of the secretion of reproductive hormones,^70^ leading to lower semen quality.^71,72^ As mentioned previously, epidemiological studies have found associations between SRH and altered circulating reproductive hormones.^24,25,26^

### Strengths and Limitations

The current study has several strengths. First, 2 semen samples at different times were collected from each participant for measurements, which can reduce the intra-individual variation and accurately reflect semen quality over time.^28^ Second, our study had a large sample size and consideration of multi-item scales on SRH to detect more effective indicators of semen quality. Third, the modifying effects of age and BMI on the association between SRH and semen quality were explored.

However, this study also has some limitations. First, although the participants were not aware of their semen quality parameters as they reported the SRH, the cross-sectional data makes it difficult to draw causal inferences. Second, the participants were those men undergoing ART treatment, which included more men with infertility or subfertility. However, our results were robust after excluding the participants with reproductive diseases. Nevertheless, participants with other underlying diseases may bias semen quality, which may limit the generalizability of the results to other populations. Third, information on SRH was collected only once, and thus the possibility of exposure misclassification cannot be ruled out. However, studies have indicated that SRH is sensitive to mental and physical changes in health and shows high reliability.^30^ Additionally, some confounding factors, including dietary habits, associated with semen quality and SRH may bias the observed results.

## Conclusions

In this cross-sectional study, we found that poorer SRH was associated with lower semen quality among men undergoing ART. Specifically, it seemed that reproductive-related physical SRH had the greatest association with semen quality. These suggest that SRH, particularly reproductive-related physical health, may be a valid indicator of semen quality, which is of significant public health and clinical implications. Our findings warrant further confirmation using different populations and prospective studies.

## Notes

Supplement 1. **eTable 1.** Characteristics of the Included Population and the Whole Population **eTable 2.** Comparisons of Semen Quality Among 4 Components of Self-Rated Health **eTable 3.** Percentage Variations and Regression Coefficients for Semen Quality Parameters Associated With Self-Rated Health Based on Linear Mixed-Effects Models Among 1262 Male Participants **eTable 4.** Percentage Variations and Regression Coefficients for Semen Quality Parameters Associated With Self-Rated Health Based on Linear Mixed-Effects Models After Excluding Men With Reproductive Diseases **eTable 5.** Percentage Variations and Regression Coefficients for Semen Quality Parameters Associated With Self-Rated Health Based on Linear Mixed-Effects Models After Excluding Men With Reproductive Diseases and Medication For Spermatogenesis **eTable 6.** Percentage Variations and Regression Coefficients for Semen Quality Parameters Associated With Self-Rated Health Stratified by Age Based on Linear Mixed-Effects Models **eTable 7.** Percentage Variations and Regression Coefficients for Semen Quality Parameters Associated With Self-Rated Health Stratified by BMI Based on Linear Mixed-Effects Models **eTable 8.** Correlation Coefficients of 4 Items of Self-Rated Health Based on Spearman Correlation Analysis **eFigure.** Selection of Potential Confounders in Self-Rated Health and Semen Quality by Directed Acyclic Graphs

Supplement 2.
Data Sharing Statement
