# Error in Author Affiliations

**DOI:** 10.1001/jamanetworkopen.2024.2922

**Published:** 2024-02-21

The Original Investigation titled “Self-Rated Health and Semen Quality in Men Undergoing Assisted Reproductive Technology,”^1^ published January 30, 2024, had an error in an author’s affiliation. Dr Zeng’s affiliation in the Corresponding Authors section should have read “Department of Occupational and Environmental Health, School of Public Health, Tongji Medical College, Huazhong University of Science and Technology, No.13, Hangkong Road, Wuhan, Hubei 430030, China.” This article has been corrected.^1^
