# Supplementary material for: Self-Rated Health and Semen Quality in Men Undergoing Assisted Reproductive Technology
Source: JAMA Netw Open. 2024 Jan 30;7(1):e2353877. doi: 10.1001/jamanetworkopen.2023.53877 (PMC10828918; doi:10.1001/jamanetworkopen.2023.53877)
Supplement: Supplement 1. — eTable 1. Characteristics of the Included Population and the Whole Population eTable 2. Comparisons of Semen Quality Among 4 Components of Self-Rated Health eTable 3. Percentage Variations and Regression Coefficients for Semen Quality Parameters Associated With Self-Rated Health Based on Linear Mixed-Effects Models Among 1262 Male Participants eTable 4. Percentage Variations and Regression Coefficients for Semen Quality Parameters Associated With Self-Rated Health Based on Linear Mixed-Effects Models After Excluding Men With Reproductive Diseases eTable 5. Percentage Variations and Regression Coefficients for Semen Quality Parameters Associated With Self-Rated Health Based on Linear Mixed-Effects Models After Excluding Men With Reproductive Diseases and Medication For Spermatogenesis eTable 6. Percentage Variations and Regression Coefficients for Semen Quality Parameters Associated With Self-Rated Health Stratified by Age Based on Linear Mixed-Effects Models eTable 7. Percentage Variations and Regression Coefficients for Semen Quality Parameters Associated With Self-Rated Health Stratified by BMI Based on Linear Mixed-Effects Models eTable 8. Correlation Coefficients of 4 Items of Self-Rated Health Based on Spearman Correlation Analysis eFigure. Selection of Potential Confounders in Self-Rated Health and Semen Quality by Directed Acyclic Graphs [file jamanetwopen-e2353877-s001.pdf]

Supplemental Online Content

Liu XY, Deng YL, Chen PP, et al. Self-rated health and semen quality in men undergoing assisted reproductive technology. *JAMA Netw Open.* 2024;7(1):e2353877. doi:10.1001/jamanetworkopen.2023.53877

eTable 1. Characteristics of the Included Population and the Whole Population

eTable 2. Comparisons of Semen Quality Among 4 Components of Self-Rated Health

eTable 3. Percentage Variations and Regression Coefficients for Semen Quality Parameters Associated With Self-Rated Health Based on Linear Mixed-Effects Models Among 1262 Male Participants

eTable 4. Percentage Variations and Regression Coefficients for Semen Quality Parameters Associated With Self-Rated Health Based on Linear Mixed-Effects Models After Excluding Men With Reproductive Diseases

eTable 5. Percentage Variations and Regression Coefficients for Semen Quality Parameters Associated With Self-Rated Health Based on Linear Mixed-Effects Models After Excluding Men With Reproductive Diseases and Medication For Spermatogenesis

eTable 6. Percentage Variations and Regression Coefficients for Semen Quality Parameters Associated With Self-Rated Health Stratified by age Based on Linear Mixed-Effects Models

eTable 7. Percentage Variations and Regression Coefficients for Semen Quality Parameters Associated With Self-Rated Health Stratified by BMI Based on Linear Mixed-Effects Models

eTable 8. Correlation Coefficients of 4 Items of Self-Rated Health Based on Spearman Correlation Analysis

eFigure. Selection of Potential Confounders in Self-Rated Health and Semen Quality by Directed Acyclic Graphs

This supplemental material has been provided by the authors to give readers additional information about their work.

© 2024 Liu XY et al. *JAMA Network Open.*

eTable 1. Characteristics of the Included Population and the Whole Population [Mean±SD or n (%)]

Characteristics	Included population (N=1262)	Whole population (N=2045)	P[a]
Age at recruitment (years)	32.79 ± 5.25	32.58 ± 5.18	0.28
<30	353 (28.0)	488 (23.9)	
≥30	909 (72.0)	1136 (55.6)	
Ethnicity			0.92
Han	1215 (96.3)	1967 (96.2)	
Other[b]	47 (3.7)	78 (3.8)	
BMI (kg/m^2)	24.37 ± 3.68	24.40 ± 3.64	0.59
<18.5	36 (2.9)	50 (2.4)	
18.5-24	546 (43.3)	712 (34.8)	
≥24	680 (53.9)	861 (42.1)	
Smoking status			0.73
Never-smoker	542 (42.9)	904 (44.2)	
Former smoker	190 (15.1)	310 (15.2)	
Current smoker	530 (42.0)	831 (40.6)	
Alcohol use			0.52
No	1006 (79.7)	1611 (78.8)	
Yes	256 (20.3)	434 (21.2)	
Educational level			0.91
Less than high school	413 (32.7)	673 (32.9)	
High school and above	849 (67.3)	1372 (67.1)	
Household income (yuan/month)			0.85
≤3000	133 (10.5)	216 (10.6)	
3000-10,000	888 (70.4)	1422 (69.5)	
≥10,000	241 (19.1)	407 (19.9)	
Abstinence time at first measurement (days)			0.58
<3	157 (12.4)	179 (8.8)	
3-5	863 (68.4)	1082 (52.9)	
>5	232 (18.4)	306 (15.0)	
Abstinence time at second measurement (days)			0.02
<3	96 (7.6)	128 (6.3)	
3-5	947 (75.0)	1154 (56.4)	
>5	176 (13.9)	286 (14.0)	
Reproductive-related physical health			0.91
Poor	606 (48.0)	997 (48.8)	
Good	456 (36.1)	725 (35.5)	

© 2024 Liu XY et al. *JAMA Network Open.*

Very good	200 (15.8)	323 (15.8)	
Reproductive-related mental health			0.87
Poor	501 (39.7)	819 (40.0)	
Good	512 (40.6)	838 (41.0)	
Very good	249 (19.7)	388 (19.0)	
Overall physical health			0.71
Poor	538 (42.6)	854 (41.8)	
Good	510 (40.4)	856 (41.9)	
Very good	214 (17.0)	335 (16.4)	
Overall mental health			0.74
Poor	414 (32.8)	659 (32.2)	
Good	568 (45.0)	948 (46.4)	
Very good	280 (22.2)	438 (21.4)	

Abbreviations: BMI: body mass index; SD: standard deviation.10 missing abstinence time at first measurement and 43 missing abstinence time at second measurement in the included population. 421 missing age, 422 missing BMI, 478 missing abstinence time at first measurement, and 477 missing abstinence time at second measurement in the whole population.

[a] *P*-values between the included population and the whole population.

[b] Others included Bai, Dong, Hui, Man, Mongolian, Miao, Tujia, Uighur, Yao, Yi, and Zhuang ethnic groups.

© 2024 Liu XY et al. *JAMA Network Open.*

eTable 2. Comparisons of Semen Quality Among 4 Components of Self-Rated Health[a]

Self-rated health	First measurement [Median (25–75 percentiles)][b]				Second measurement [Median (25–75 percentiles)][c]			
	Sperm concentration (million/mL)	Sperm count (million/ejaculate)	Sperm motility (% motile)	Sperm progressive motility (% motile)	Sperm concentration (million/mL)	Sperm count (million/ejaculate)	Sperm motility (% motile)	Sperm progressive motility (% motile)
Reproductive-related physical health								
Poor	42.1 (23.8-68.9)	147.3 (147.3-246.0)	47.3 (33.3-62.1)	44.7 (30.5-59.5)	38.0 (20.0-64.8)	105.0 (54.0-196.0)	41.0 (25.0-54.0)	38.0 (22.0-51.0)
Good	51.9 (30.1-77.8)	184.1 (107.3-279.8)	55.4 (41.0-67.2)	51.4 (37.9-64.3)	46.0 (25.0-73.0)	140.0 (72.0-210.0)	46.0 (33.0-57.0)	43.0 (30.0-54.0)
Very good	51.2 (31.3-86.8)	203.6 (113.7-284.4)	58.1 (45.5-71.6)	54.9 (43.4-68.8)	50.0 (31.0-92.0)	132.0 (82.0-234.0)	50.0 (37.0-61.0)	46.0 (33.0-57.0)
Reproductive-related mental health								
Poor	43.3 (24.3-70.5)	152.4 (81.9-250.7)	48.2 (33.7-63.4)	45.5 (30.8-60.3)	38.0 (20.0-63.0)	112.0 (55.0-196.0)	41.0 (25.0-54.0)	38.0 (22.0-51.0)
Good	49.7 (28.8-76.3)	176.6 (103.6-266.7)	52.9 (40.1-65.8)	49.7 (37.1-62.8)	46.0 (25.0-73.0)	130.0 (66.0-215.0)	45.0 (32.0-57.0)	43.0 (29.0-54.0)
Very good	49.5 (29.4-85.3)	194.3 (101.6-280.8)	57.3 (43.4-70.6)	54.0 (41.6-67.4)	45.0 (28.0-85.0)	132.0 (72.0-222.0)	49.0 (36.0-60.0)	45.0 (32.0-57.0)
Overall physical health								

© 2024 Liu XY et al. *JAMA Network Open.*

Poor	44.0 (24.5-72.4)	151.7 (81.0-250.4)	48.9 (35.9-65.0)	45.7 (32.8-61.7)	40.0 (21.0-64.0)	110.5 (55.0-195.0)	43.0 (27.0-55.0)	40.0 (24.0-53.0)
Good	48.9 (28.4-77.5)	185.4 (107.3-272.9)	53.4 (40.0-53.4)	49.8 (36.4-62.9)	46.0 (25.0-74.0)	135.0 (65.6-223.0)	45.0 (32.0-57.0)	42.0 (28.5-56.0)
Very good	49.2 (29.7-79.1)	185.2 (103.9-265.8)	55.2 (43.0-69.1)	50.9 (41.2-66.7)	44.0 (27.5-83.5)	130.0 (71.8-215.5)	45.0 (30.0-60.0)	41.0 (28.0-56.0)
Overall mental health								
Poor	43.9 (24.4-72.4)	151.1 (79.0-254.1)	48.4 (35.0-65.0)	45.7 (31.7-61.7)	39.0 (20.0-63.0)	111.0 (54.0-193.5)	42.0 (26.0-55.5)	39.0 (22.5-52.5)
Good	47.8 (28.7-75.5)	180.1 (103.7-265.2)	52.9 (39.7-66.2)	49.0 (36.2-63.0)	45.0 (25.0-73.0)	130.0 (65.0-216.0)	45.0 (30.0-57.0)	42.0 (26.0-54.0)
Very good	48.8 (26.5-79.4)	173.4 (95.3-271.4)	54.8 (42.3-54.8)	51.6 (40.6-66.0)	44.0 (26.0-82.0)	130.0 (69.0-219.0)	46.0 (34.0-59.0)	44.0 (31.0-55.0)

[a] *P* values were estimated using analysis of variance to examine differences in semen quality parameters among the three groups in the four components of self-rated health. Sperm concentration and count were natural-log transformed and all *P* values were ≤0.001.

[b] 17 missing sperm progressive motility in the first measurement.

[c] 2 missing sperm progressive motility and sperm count and 1 missing sperm concentration in the second measurement.

© 2024 Liu XY et al. *JAMA Network Open.*

eTable 3. Percentage Variations and Regression Coefficients for Semen Quality Parameters Associated With Self-Rated Health Based on Linear Mixed-Effects Models Among 1262 Male Participants

Self-rated health status	Sperm concentration (percentage variation)[b]	Sperm count (percentage variation)[b]	Sperm motility (percent motile)	Sperm progressive motility (percent motile)
**Reproductive-related physical health**				
Crude model				
Very good	Reference	Reference	Reference	Reference
Good	-10.39 (-20.19, 0.61)	-6.85 (-17.75, 5.49)	-3.77 (-6.50, -1.04)	-3.96 (-6.65, -1.27)
Poor	-24.72 (-32.65, -15.85)	-25.11 (-33.55, -15.59)	-9.64 (-12.27, -7.02)	-9.46 (-12.04, -6.87)
P_{trend}	<0.001	<0.001	<0.001	<0.001
Adjusted model[a]				
Very good	Reference	Reference	Reference	Reference
Good	-10.19 (-20.00, 0.81)	-6.96 (-17.76, 5.27)	-3.43 (-6.16, -0.70)	-3.64 (-6.32, -0.96)
Poor	-24.78 (-32.71, -15.93)	-25.61 (-33.95, -16.22)	-9.38 (-12.01, -6.76)	-9.24 (-11.82, -6.66)
P_{trend}	<0.001	<0.001	<0.001	<0.001
**Reproductive-related mental health**				
Crude model				
Very good	Reference	Reference	Reference	Reference
Good	-7.24 (-16.58, 3.14)	-5.38 (-15.59, 6.06)	-3.02 (-5.53, -0.50)	-2.90 (-5.37, -0.42)
Poor	-18.44 (-26.67, -9.27)	-18.52 (-27.33, -8.63)	-7.54 (-10.07, -5.02)	-7.16 (-9.64, -4.68)
P_{trend}	<0.001	<0.001	<0.001	<0.001
Adjusted model[a]				
Very good	Reference	Reference	Reference	Reference
Good	-7.44 (-16.75, 2.90)	-5.06 (-15.23, 6.33)	-3.04 (-5.55, -0.54)	-2.93 (-5.40, -0.47)
Poor	-18.88 (-27.07, -9.77)	-18.84 (-27.58, -9.06)	-7.52 (-10.04, -5.00)	-7.19 (-9.67, -4.71)

© 2024 Liu XY et al. *JAMA Network Open.*

P_{trend}	<0.001	<0.001	<0.001	<0.001	
**Overall physical health**					
Crude model					
Very good	Reference	Reference	Reference	Reference	
Good	-4.06 (-14.22, 7.31)	-1.42 (-12.59, 11.17)	-1.12 (-3.80, 1.56)	-1.36 (-3.99, 1.27)	
Poor	-15.35 (-24.25, -5.40)	-17.35 (-26.64, -6.88)	-4.39 (-7.04, -1.74)	-4.18 (-6.79, -1.57)	
P_{trend}	0.001	<0.001	<0.001	<0.001	<0.0
Adjusted model[a]					
Very good	Reference	Reference	Reference	Reference	
Good	-3.90 (-14.07, 7.47)	-1.16 (-12.28, 11.37)	-0.87 (-3.53, 1.80)	-1.14 (-3.76, 1.47)	
Poor	-14.67 (-23.62, -4.66)	-17.19 (-26.43, -6.79)	-4.31 (-6.95, -1.67)	-4.15 (-6.75, -1.56)	
P_{trend}	0.001	<0.001	<0.001	<0.001	
**Overall mental health**					
Crude model					
Very good	Reference	Reference	Reference	Reference	
Good	-2.25 (-11.60, 8.09)	-0.87 (-11.01, 10.43)	-2.07 (-4.46, 0.33)	-2.23 (-4.58, 0.13)	
Poor	-13.05 (-21.84, -3.28)	-16.47 (-25.50, -6.36)	-5.32 (-7.85, -2.78)	-5.04 (-7.53, -2.54)	
P_{trend}	0.01	0.001	<0.001	<0.001	
Adjusted model[a]					
Very good	Reference	Reference	Reference	Reference	
Good	-2.99 (-12.26, 7.25)	-1.52 (-11.52, 9.60)	-2.07 (-4.45, 0.32)	-2.22 (-4.57, 0.13)	
Poor	-13.56 (-22.28, -3.85)	-17.62 (-26.45, -7.72)	-5.37 (-7.90, -2.84)	-5.10 (-7.59, -2.62)	
P_{trend}	0.01	<0.001	<0.001	<0.001	

[a] Adjusted for age (continuous), BMI (continuous), alcohol use (ever vs. never), education (≥high school vs. <high school), abstinence time (3–5 and >5 vs. <3 days), income (3,000–10,000 and ≥10,000 vs. ≤3,000 yuan/month), and smoking status (current and former vs.never-smoker).

[b] Sperm concentration and count were natural-log transformed and back-transformed {100*[exp(beta)-1]} to obtain percent changes.

© 2024 Liu XY et al. *JAMA Network Open.*

eTable 4. Percentage Variations and Regression Coefficients for Semen Quality Parameters Associated With Self-Rated Health Based on Linear Mixed-Effects Models After Excluding Men With Reproductive Diseases (N=1119)

Self-rated health status	Sperm concentration (percentage variation)[b]	Sperm count (percentage variation)[b]	Sperm motility (percent motile)	Sperm progressive motility (percent motile)
**Reproductive-related physical health**				
Crude model				
Very good	Reference	Reference	Reference	Reference
Good	-10.15 (-20.21, 1.19)	-7.34 (-18.44, 5.27)	-4.09 (-6.88, -1.29)	-4.17 (-6.96, -1.38)
Poor	-22.60 (-31.06, -13.11)	-21.99 (-31.1, -11.67)	-9.59 (-12.31, -6.86)	-9.27 (-11.99, -6.56)
P_{trend}	<0.001	<0.001	<0.001	<0.001
Adjusted model[a]				
Very good	Reference	Reference	Reference	Reference
Good	-9.93 (-20.02, 1.43)	-7.50 (-18.50, 4.99)	-3.86 (-6.67, -1.06)	-4.03 (-6.79, -1.27)
Poor	-22.79 (-31.23, -13.31)	-22.65 (-31.63, -12.49)	-9.41 (-12.14, -6.68)	-9.10 (-11.79, -6.41)
P_{trend}	<0.001	<0.001	0.001	<0.001
**Reproductive-related mental health**				
Crude model				
Very good	Reference	Reference	Reference	Reference
Good	-8.17 (-17.64, 2.40)	-6.42 (-16.75, 5.19)	-3.33 (-5.90, -0.75)	-3.27 (-5.84, -0.71)
Poor	-17.12 (-25.79, -7.44)	-16.29 (-25.65, -5.75)	-7.69 (-10.30, -5.08)	-7.23 (-9.83, -4.63)
P_{trend}	0.001	0.002	<0.001	<0.001
Adjusted model[a]				
Very good	Reference	Reference	Reference	Reference
Good	-8.25 (-17.71, 2.31)	-6.16 (-16.45, 5.39)	-3.45 (-6.03, -0.87)	-3.30 (-5.84, -0.76)
Poor	-17.51 (-26.14, -7.87)	-16.64 (-25.91, -6.21)	-7.65 (-10.27, -5.03)	-7.16 (-9.74, -4.58)

© 2024 Liu XY et al. *JAMA Network Open.*

P_{trend}	<0.001	0.001	<0.001	<0.001
**Overall physical health**				
Crude model				
Very good	Reference	Reference	Reference	Reference
Good	-5.97 (-16.27, 5.60)	-3.77 (-15.02, 8.97)	-1.60 (-4.37, 1.16)	-1.89 (-4.64, 0.87)
Poor	-14.66 (-24.01, -4.16)	-15.69 (-25.55, -4.53)	-4.91 (-7.68, -2.15)	-4.54 (-7.29, -1.78)
P_{trend}	0.004	0.002	<0.001	0.001
Adjusted model[a]				
Very good				
Good	-5.73 (-16.04, 5.85)	-3.78 (-14.94, 8.86)	-1.39 (-4.15, 1.37)	-1.57 (-4.29, 1.15)
Poor	-14.02 (-23.42, -3.45)	-15.82 (-25.59, -4.77)	-4.84 (-7.60, -2.08)	-4.55 (-7.27, -1.83)
P_{trend}	0.01	0.002	<0.001	<0.001
**Overall mental health**				
Crude model				
Very good	Reference	Reference	Reference	Reference
Good	-2.84 (-12.44, 7.81)	-0.49 (-10.98, 11.23)	-2.08 (-4.55, 0.39)	-2.19 (-4.65, 0.27)
Poor	-11.55 (-20.88, -1.11)	-13.62 (-23.34, -2.66)	-5.5 (-8.15, -2.85)	-5.05 (-7.69, -2.41)
P_{trend}	0.02	0.01	<0.001	<0.001
Adjusted model[a]				
Very good	Reference	Reference	Reference	Reference
Good	-3.71 (-13.22, 6.84)	-1.68 (-11.97, 9.82)	-2.05 (-4.52, 0.43)	-2.14 (-4.58, 0.29)
Poor	-12.17 (-21.44, -1.81)	-15.03 (-24.54, -4.33)	-5.48 (-8.14, -2.83)	-5.07 (-7.68, -2.45)
P_{trend}	0.02	0.004	<0.001	<0.001

[a] Adjusted for age (continuous), BMI (continuous), alcohol use (ever vs. never), education (≥high school vs. <high school), abstinence time (3–5 and >5 vs. <3 days), income (3,000–10,000 and ≥10,000 vs. ≤3,000 yuan/month), and smoking status (current and former

© 2024 Liu XY et al. *JAMA Network Open.*

vs.never-smoker).

[b] Sperm concentration and count were natural-log transformed and back-transformed {100*[exp(beta)-1]} to obtain percentage variations.

© 2024 Liu XY et al. *JAMA Network Open.*

eTable 5. Percentage Variations and Regression Coefficients for Semen Quality Parameters Associated With Self-Rated Health Based on Linear Mixed-Effects Models After Excluding Men With Reproductive Diseases and Medication For Spermatogenesis (N=974)

Self-rated health status	Sperm concentration (percentage variation)[b]	Sperm count (percentage variation)[b]	Sperm motility (percent motile)	Sperm progressive motility (percent motile)
**Reproductive-related physical health**				
Crude model				
Very good	Reference	Reference	Reference	Reference
Good	-8.90 (-19.00, 2.45)	-6.88 (-17.89, 5.61)	-3.45 (-6.21, -0.69)	-3.72 (-6.45, -0.99)
Poor	-16.35 (-25.53, -6.03)	-15.27 (-25.19, -4.02)	-7.51 (-10.25, -4.78)	-7.36 (-10.07, -4.66)
P_{trend}	0.002	0.01	<0.001	<0.001
Adjusted model[a]				
Very good	Reference	Reference	Reference	Reference
Good	-8.73 (-18.86, 2.66)	-6.95 (-17.84, 5.39)	-3.36 (-6.14, -0.58)	-3.65 (-6.40, -0.91)
Poor	-16.53 (-25.70, -6.23)	-16.11 (-25.84, -5.11)	-7.49 (-10.24, -4.74)	-7.39 (-10.11, -4.68)
P_{trend}	0.002	0.003	<0.001	<0.001
**Reproductive-related mental health**				
Crude model				
Very good	Reference	Reference	Reference	Reference
Good	-6.67 (-16.29, 4.06)	-4.93 (-15.39, 6.82)	-2.76 (-5.33, -0.18)	-2.82 (-5.37, -0.28)
Poor	-11.25 (-20.68, -0.69)	-9.28 (-19.57, 2.33)	-5.54 (-8.19, -2.88)	-5.27 (-7.90, -2.64)
P_{trend}	0.04	0.11	<0.001	<0.001
Adjusted model[a]				
Very good	Reference	Reference	Reference	Reference
Good	-6.86 (-16.47, 3.86)	-4.84 (-15.21, 6.80)	-3.05 (-5.63, -0.46)	-3.11 (-5.66, -0.56)
Poor	-11.47 (-20.89, -0.93)	-9.59 (-19.74, 1.85)	-5.65 (-8.32, -2.98)	-5.47 (-8.10, -2.84)

© 2024 Liu XY et al. *JAMA Network Open.*

P_{trend}	0.04	0.09	<0.001	<0.001
**Overall physical health**				
Crude model				
Very good	Reference	Reference	Reference	Reference
Good	-3.55 (-14.07, 8.25)	-1.26 (-12.73, 11.71)	-0.60 (-3.33, 2.14)	-0.91 (-3.61, 1.8)
Poor	-8.72 (-18.77, 2.57)	-9.30 (-19.94, 2.75)	-3.63 (-6.40, -0.87)	-3.44 (-6.17, -0.71)
P_{trend}	0.10	0.08	0.003	0.006
Adjusted model[a]				
Very good	Reference	Reference	Reference	Reference
Good	-3.27 (-13.82, 8.56)	-1.21 (-12.56, 11.62)	-0.51 (-3.26, 2.24)	-0.86 (-3.57, 1.85)
Poor	-8.21 (-18.30, 3.12)	-9.66 (-20.12, 2.17)	-3.64 (-6.40, -0.87)	-3.50 (-6.23, -0.76)
P_{trend}	0.12	0.06	0.003	0.005
**Overall mental health**				
Crude model				
Very good	Reference	Reference	Reference	Reference
Good	-2.06 (-11.84, 8.79)	0.92 (-9.81, 12.93)	-1.56 (-4.05, 0.93)	-1.87 (-4.33, 0.6)
Poor	-5.61 (-15.82, 5.84)	-7.48 (-18.15, 4.57)	-4.14 (-6.86, -1.43)	-4.02 (-6.70, -1.34)
P_{trend}	0.31	0.18	0.002	0.003
Adjusted model[a]				
Very good	Reference	Reference	Reference	Reference
Good	-3.05 (-12.73, 7.71)	-0.69 (-11.14, 10.99)	-1.69 (-4.20, 0.81)	-2.01 (-4.48, 0.46)
Poor	-6.42 (-16.55, 4.94)	-9.24 (-19.75, 2.64)	-4.19 (-6.92, -1.46)	-4.13 (-6.82, -1.44)
P_{trend}	0.25	0.10	0.002	0.003

[a] Adjusted for age (continuous), BMI (continuous), alcohol use (ever vs. never), education (≥high school vs. <high school), abstinence time (3–5 and >5 vs. <3 days), income (3,000–10,000 and ≥10,000 vs. ≤3,000 yuan/month), and smoking status (current and former vs.never-

© 2024 Liu XY et al. *JAMA Network Open.*

smoker).

[b] Sperm concentration and count were natural-log transformed and back-transformed {100*[exp(beta)-1]} to obtain percentage variations.

© 2024 Liu XY et al. *JAMA Network Open.*

eTable 6. Percentage Variations and Regression Coefficients for Semen Quality Parameters Associated With Self-Rated Health Stratified by age Based on Linear Mixed-Effects Models (N=1262)

Self-rated health status[a]	Sperm concentration (percentage variation)[b]		Sperm count (percentage variation)[b]		Sperm motility (percent motile)		Sperm progressive motility (percent motile)	
	<30 years (n=705)	≥30 years (n=1818)	<30 years (n=705)	≥30 years (n=1817)	<30 years (n=706)	≥30 years (n=1818)	<30 years (n=703)	≥30 years (n=1802)
Reproductive-related physical health								
Very good	Reference	Reference	Reference	Reference	Reference	Reference	Reference	Reference
Good	-9.38 (-27.24, 12.87)	-9.13 (-20.68, 4.09)	-8.81 (-28.24, 15.88)	-5.03 (-17.79, 9.71)	-1.96 (-7.13, 3.2)	-4.00 (-7.21, -0.78)	-2.36 (-7.45, 2.73)	-4.10 (-7.25, -0.94)
Poor	-27.61 (-41.33, -10.68)	-22.19 (-31.77, -11.27)	-26.95 (-41.93, -8.12)	-24.37 (-34.21, -13.05)	-9.78 (-14.72, -4.83)	-8.95 (-12.05, -5.84)	-9.72 (-14.59, -4.85)	-8.84 (-11.89, -5.79)
P_{trend}	0.001	<0.001	0.002	<0.001	<0.001	<0.001	<0.001	<0.001
P for interaction	0.40		0.83		0.43		0.49	
Reproductive-related mental health								
Very good	Reference	Reference	Reference	Reference	Reference	Reference	Reference	Reference
Good	-2.51 (-20.19, 19.08)	-8.59 (-19.30, 3.54)	-2.75 (-21.81, 20.95)	-5.84 (-17.54, 7.51)	-1.31 (-6.04, 3.43)	-3.87 (-6.82, -0.91)	-0.97 (-5.62, 3.68)	-3.82 (-6.72, -0.92)
Poor	-16.69 (-31.86, 1.84)	-18.42 (-28.05, -7.51)	-16.31 (-32.77, 4.18)	-19.64 (-29.70, -8.15)	-7.32 (-12.08, -2.57)	-7.60 (-10.58, -4.63)	-6.86 (-11.54, -2.18)	-7.31 (-10.24, -4.39)
P_{trend}	0.05	0.001	0.08	<0.001	0.001	<0.001	0.002	<0.001
P for interaction	0.98		0.79		0.98		0.99	

© 2024 Liu XY et al. *JAMA Network Open.*

Overall physical health								
Very good	Reference	Reference	Reference	Reference	Reference	Reference	Reference	Reference
Good	-1.11 (-20.04, 22.28)	-5.07 (-16.74, 8.23)	-0.80 (-21.22, 24.92)	-1.81 (-14.60, 12.90)	1.45 (-3.62, 6.52)	-2.17 (-5.30, 0.96)	1.46 (-3.53, 6.44)	-2.51 (-5.58, 0.57)
Poor	-17.80 (-33.45, 1.54)	-13.01 (-23.62, -0.93)	-21.66 (-37.71, -1.48)	-15.06 (-26.03, -2.46)	-3.99 (-9.03, 1.06)	-4.40 (-7.50, -1.29)	-3.57 (-8.54, 1.39)	-4.31 (-7.36, -1.26)
P_{trend}	0.03	0.02	0.01	0.01	0.04	0.004	0.05	0.005
P for interaction	0.40		0.40		0.71		0.76	
Overall mental health								
Very good	Reference	Reference	Reference	Reference	Reference	Reference	Reference	Reference
Good	3.24 (-14.68, 24.92)	-4.34 (-15.01, 7.67)	-2.47 (-20.73, 19.98)	-0.43 (-12.19, 12.90)	-0.94 (-5.47, 3.58)	-2.46 (-5.28, 0.37)	-0.93 (-5.39, 3.54)	-2.67 (-5.44, 0.09)
Poor	-9.50 (-25.89, 10.52)	-13.81 (-24.01, -2.24)	-16.29 (-32.63, 4.01)	-16.89 (-27.30, -4.99)	-6.04 (-10.79, -1.30)	-4.78 (-7.79, -1.78)	-5.28 (-9.96, -0.60)	-4.74 (-7.69, -1.79)
P_{trend}	0.27	0.02	0.09	0.003	0.01	0.002	0.02	0.002
P for interaction	0.82		0.98		0.60		0.77	

[a] Adjusted for BMI (continuous), alcohol use (ever vs. never), education (⩾high school vs. <high school), abstinence time (3 – 5 and >5 vs. <3 days), income (3,000 – 10,000 and ⩾10,000 vs. ⩽3,000 yuan/month), and smoking status (current and former vs.never-smoker).

[b] Sperm concentration and count were natural-log transformed and back-transformed {100*[exp(beta)-1]} to obtain percentage variation.

© 2024 Liu XY et al. *JAMA Network Open.*

eTable 7. Percentage Variations and Regression Coefficients for Semen Quality Parameters Associated With Self-Rated Health Stratified by BMI Based on Linear Mixed-Effects Models (N=1262)

Self-rated health status[a]	Sperm concentration (percentage variation)[b]		Sperm count (percentage variation)[b]		Sperm motility (percent motile)		Sperm progressive motility(percent motile)	
	<24kg/m^2 (n=1163)	≥24 kg/m^2 (n=1360)	<24 kg/m^2 (n=1163)	≥24 kg/m^2 (n=1360)	<24 kg/m^2 (n=1164)	≥24kg/m^2 (n=1360)	<24 kg/m^2 (n=1156)	≥24 kg/m^2 (n=1348)
Reproductive-related physical health								
Very good	Reference	Reference	Reference	Reference	Reference	Reference	Reference	Reference
Good	-14.59 (-28.11, 1.47)	-7.10 (-20.52, 8.59)	-11.98 (-26.82, 5.88)	-2.66 (-17.53, 14.88)	-3.59 (-7.68, 0.50)	-3.52 (-7.18, 0.13)	-3.90 (-7.91, 0.11)	-3.69 (-7.29, -0.09)
Poor	-32.63 (-43.01, -20.34)	-18.07 (-29.42, -4.89)	-33.36 (-44.31, -20.25)	-19.03 (-30.89, -5.14)	-10.53 (-14.50, -6.55)	’-8.51 (-12.00, -5.01)	-10.12 (-14.01, -6.22)	-8.59 (-12.03, -5.14)
P_{trend}	<0.001	0.004	<0.001	0.002	<0.001	<0.001	<0.001	<0.001
P for interaction	0.08		0.12		0.40		0.56	
Reproductive-related mental health								
Very good	Reference	Reference	Reference	Reference	Reference	Reference	Reference	Reference
Good	-14.34 (-27.02, 0.54)	-2.63 (-15.52, 12.22)	-11.52 (-25.47, 5.04)	-0.02 (-14.04, 16.29)	-4.46 (-8.27, -0.65)	-2.32 (-5.66, 1.01)	-4.19 (-7.92, -0.46)	-2.35 (-5.65, 0.94)
Poor	-27.75 (-38.60, -15.00)	-11.73 (-23.34, 1.63)	-28.68 (-40.08, -15.11)	-10.70 (-23.14, 3.75)	-9.04 (-12.91, -5.17)	-6.51 (-9.82, -3.20)	-8.37 (-12.16, -4.58)	-6.48 (-9.74, -3.21)
P_{trend}	<0.001	0.06	<0.001	0.09	<0.001	<0.001	<0.001	<0.001
P for interaction	0.08		0.05		0.43		0.60	

© 2024 Liu XY et al. *JAMA Network Open.*

Overall physical health								
Very good	Reference	Reference	Reference	Reference	Reference	Reference	Reference	Reference
Good	-13.67 (-27.21, 2.39)	2.86 (-11.36, 19.35)	-11.36 (-26.14, 6.37)	6.71 (-8.89, 24.98)	-3.45 (-7.54, 0.64)	0.62 (-2.90, 4.13)	-3.66 (-7.67, 0.34)	0.27 (-3.20, 3.74)
Poor	-25.96 (-37.55, -12.21)	-5.30 (-18.20, 9.64)	-28.61 (-40.49, -14.36)	-7.09 (-20.47, 8.55)	-6.44 (-10.52, -2.35)	-3.05 (-6.51, 0.41)	-5.98 (-9.97, -1.98)	-3.16 (-6.58, 0.25)
P_{trend}	<0.001	0.31	<0.001	0.16	0.001	0.03	0.003	0.03
P for interaction	0.05		0.04		0.38		0.52	
Overall mental health								
Very good	Reference	Reference	Reference	Reference	Reference	Reference	Reference	Reference
Good	-4.56 (-18.00, 11.08)	-2.72 (-14.98, 11.31)	-3.76 (-18.16, 13.18)	0.19 (-13.16, 15.60)	-2.45 (-6.07, 1.16)	-2.11 (-5.30, 1.07)	-2.68 (-6.22, 0.87)	-2.23 (-5.37, 0.92)
Poor	-17.74 (-29.83, -3.57)	-9.62 (-21.76, 4.42)	-22.08 (-34.25, -7.65)	-13.25 (-25.57, 1.12)	-6.18 (-9.97, -2.39)	-4.76 (-8.17, -1.35)	-5.77 (-9.48, -2.06)	-4.68 (-8.05, -1.31)
P_{trend}	0.01	0.16	0.002	0.05	0.001	0.01	0.002	0.01
P for interaction	0.41		0.34		0.70		0.80	

[a] Adjusted for age (continuous), alcohol use (ever vs. never), education (≥high school vs. <high school), abstinence time (3 – 5 and >5 vs. <3 days), income (3,000 – 10,000 and ≥10,000 vs. ≤3,000 yuan/month), and smoking status (current and former vs.never-smoker).

[b] Sperm concentration and count were natural-log transformed and back-transformed {100*[exp(beta)-1]} to obtain percentage variation.

© 2024 Liu XY et al. *JAMA Network Open.*

eTable 8. Correlation Coefficients of 4 Items of Self-Rated Health Based on Spearman Correlation Analysis

Variable		Correlation coefficient	*P* value
Reproductive-related physical health	Reproductive-related mental health	0.78	<0.001
Reproductive-related physical health	Overall physical health	0.73	<0.001
Reproductive-related physical health	Overall mental health	0.67	<0.001
Reproductive-related mental health	Overall physical health	0.70	<0.001
Reproductive-related mental health	Overall mental health	0.81	<0.001
Overall physical health	Overall mental health	0.75	<0.001

© 2024 Liu XY et al. *JAMA Network Open.*

eFigure. Selection of Potential Confounders in Self-Rated Health and Semen Quality by Directed Acyclic Graphs

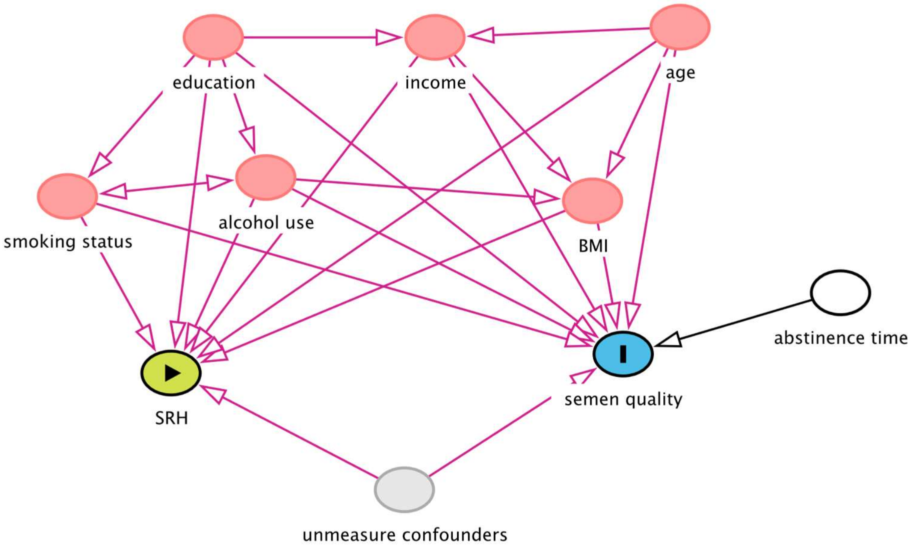

© 2024 Liu XY et al. *JAMA Network Open.*
