# Supplementary material for: Self-Rated Health and Semen Quality in Men Undergoing Assisted Reproductive Technology
Source: JAMA Netw Open. 2024 Jan 30;7(1):e2353877. doi: 10.1001/jamanetworkopen.2023.53877 (PMC10828918; doi:10.1001/jamanetworkopen.2023.53877)
Supplement: Supplement 2. — Data Sharing Statement [file jamanetwopen-e2353877-s002.pdf]

Data Sharing Statement

Liu. Self-Rated Health and Semen Quality in Men Undergoing Assisted Reproductive Technology. *JAMA Netw Open*. Published January 30, 2024.
doi:10.1001/jamanetworkopen.2023.53877

Data
Data available: No

Additional Information
Explanation for why data not available: Data will be made available to the editors of the journal for review or query upon request.
